# Use of overactive bladder anticholinergic medications associated with falls leading to emergency department visits: results from the ADRED study

**DOI:** 10.1007/s00228-023-03530-3

**Published:** 2023-06-29

**Authors:** Katja S. Just, Karen A. Schultze, Harald Dormann, Thomas Seufferlein, Ingo Gräff, Catharina Scholl, Matthias Schwab, Julia C. Stingl

**Affiliations:** 1 Institute of Clinical Pharmacology, University Hospital RWTH Aachen, Aachen, Wendlingweg 2, D-52074 Aachen, Germany; 2 Central Emergency Department, Hospital Fürth, Fürth, Germany; 3 Internal Medicine Emergency Department, Ulm University Medical Centre, Ulm, Germany; 4 Interdisciplinary Emergency Department (INZ), University Hospital of Bonn, Bonn, Germany; 5 Research Department, Federal Institute for Drugs and Medical Devices, Bonn, Germany; 6 Dr. Margarete Fischer-Bosch-Institute of Clinical Pharmacology, Stuttgart, Germany; 7 Department of Clinical Pharmacology, University of Tuebingen, Tuebingen, Germany; 8 Department of Pharmacy and Biochemistry, University of Tuebingen, Tuebingen, Germany

**Keywords:** Fall-risk increasing drugs, Older adults, Adverse drug reaction, Urologicals, Anticholinergic, Overactive bladder anticholinergic medications, Multi-medication

## Abstract

**Purpose:**

Drug intake might be a modifiable factor for the individual fall-risk of older adults, and anticholinergic properties of drugs need to be considered. This study is aimed at analyzing the association of older adults’ individual anticholinergic load with particular focus on use of overactive bladder anticholinergic medications with falls in multi-medicated patients.

**Materials and Methods:**

Cases of the prospective, observational, multi-center study on adverse drug reactions leading to emergency departments (ADRED study) between 2015 and 2018 in Germany were analyzed comparing the exposure of overactive bladder anticholinergic medications on the chance to present with a fall with patients without exposure. Logistic regression analysis was used adjusting for pre-existing conditions, drug exposure, and the individual anticholinergic burden by drug use. To this end, a combination of seven expert-based anticholinergic rating scales was used.

**Results:**

The anticholinergic burden was higher in patients with overactive bladder anticholinergic medications (median 2 [1; 3]) compared to not taking drugs of interest. Presenting with a fall was associated with overactive bladder anticholinergic medications (odds ratio (OR) 2.34 [95% confidence interval 1.14–4.82]). The use of fall-risk increasing drugs was likewise associated (OR 2.30 [1.32–4.00]). The anticholinergic burden itself seemed not to be associated with falls (OR 1.01 [0.90–1.12]).

**Conclusions:**

Although falls occur multifactorial in older adults and confounding by indication cannot be ruled out, the indication for a drug treatment should be decided with caution when other, non-pharmacological treatment options have been tried.

**German clinical trial register:**

DRKS-ID: DRKS00008979, registration date 01/11/2017.

**Supplementary Information:**

The online version contains supplementary material available at 10.1007/s00228-023-03530-3.

## Introduction

Falls are a major health issue for older adults as they can lead to disabilities [1]. Around a third of all community-dwelling older adults fall at least once a year often resulting in fractures or injuries [2]. Rates of older adults presenting with falls to the emergency department (ED) increased in the last years in the United States [3]. The risk for ED admission rises with higher age and in female gender. Preventive strategies for falls are often discussed and recommended [4].

While falls are considered to be multifactorial with factors such as motor weakness or cognitive impairment enhancing the individual fall-risk [2], the use of certain drugs is likewise discussed. Patients with high anticholinergic drug load were observed more commonly presenting with falls or fractures to the ED in Korea [5]. Hence, the risk for falls seems to increase with higher anticholinergic load as seen in retrospective data [6].

Several systematic reviews on fall-risk increasing drugs (FRIDs) exist. However, evidence usually derives from observational studies showing large heterogeneity [7]. Often, psychotropic drugs such as antipsychotics, sedatives, or antidepressants are considered fall-risk increasing. Interestingly, those drug classes often have a high anticholinergic potential such as tricyclic antidepressants. Correspondingly, psychotropic drug use is observed commonly in older adults presenting with falls to the ED in Germany [8].

Also, other drug classes such as anticholinergics or overactive bladder anticholinergic medications are considered to increase the risk for falls in older adults [9]. In lists for potentially inappropriate medications for older adults, urologicals in general are not considered as anticholinergics [10] and the impact of overactive bladder anticholinergic medications on falls of older adults is often discussed with controverse findings [7, 9].

This study is aimed at analyzing the association of older adults’ individual anticholinergic load with particular focus on use of overactive bladder anticholinergic medications with falls in multi-medicated patients.

## Materials and methods

### Study population

We analyzed data of the multicenter observational study on Adverse Drug Reactions in Emergency Departments (ADRED) (German Clinical Trial Register: DRKS00008979, registration date 01/11/2017). The ADRED study collected cases of ADRs that presented to EDs with the primary study aim to analyze the proportion of medication errors in ADRs. Patients were interrogated for their most current drug use. If appropriate, information from the most current medication records was added. All medications were documented that were seen in a timely plausible association with the ED consultation. Data were collected from December 2015 until March 2018 in four German EDs of tertiary care and teaching hospitals (Ulm, Fürth, Bonn and Stuttgart). A feasibility study showed that ADRs are seen responsible for 6.5% of ED consultations in these study centers [11]. The inclusion criteria were adult patients presenting symptoms seen in a possible, probable, or certain relation to a drug assessed with the standardized causality assessment of the WHO Uppsala Monitoring Centre. The causality assessment was conducted by the study personnel, consisting of trained physicians or pharmacists.

Patients agreed to take part in the study and provided written informed consent. The study was approved by the responsible ethical committee of the University of Bonn (202/15).

### Definition of falls

For all patients, ADR symptoms leading to or presented at ED consultation were documented. If a patient reported a fall that led to ED consultation, those cases were counted as fallers. All patients with other ADR symptoms were classified as non-fallers.

### Drug use

Several drug classes were considered as FRIDs and therefore included in our analysis [9] (Supplement 1). Drug groups were defined using the Anatomic Therapeutic Chemical classification system (ATC-classification). We focused on overactive bladder anticholinergic medications used for urinary frequency and incontinence (ATC code: G04BD).

Anticholinergic drugs were identified, and the anticholinergic burden score (ABS) was calculated according to a review combining seven anticholinergic risk scales[12] (Supplement 2). An ABS was calculated for each patient enrolled. We calculated three different versions of this score, an overall anticholinergic burden score (ABS), a score excluding urologicals (ABS-U) and one excluding drugs assessed as FRIDs (ABS-F).

### Included variables

We analyzed general information as well as factors related to the ED visit. All patients enrolled in the study were interrogated for their past medical history, and if appropriate, this information was extended by a most current documentation letter. All diseases reported were coded by an ICD-10 code and then included in the analysis. From the literature, certain conditions potentially increasing the risk for falls were identified and defined with the ICD-10 classification. These included rheumatic diseases (including arthrosis [13], gout, rheumatoid arthritis [14], and connective tissue diseases), diseases of the back (including ankylosing spondylitis[15]), soft tissue diseases (including muscular diseases [16], sarcopenia [17]), osteopathies and chondropathies (including osteoporosis), abnormalities of gait, and a tendency to fall [18]. Apart from musculoskeletal diseases, other pre-existing conditions likely to increase the risk of falling were included: dementia [19] (including Alzheimer’s disease, vascular dementia, dementia in other diseases, and unspecified dementia), Parkinson’s disease [20], multiple sclerosis [21], epilepsy [22], polyneuropathy [23], cerebral palsy and paralysis syndrome, chronic pain, atherosclerosis of arteries of extremities, and urinary incontinence. Further, affective, neurotic, stress, and somatoform disorders were regarded as one variable.

All patient cases were assessed for the mentioned pre-existing conditions and this information included in our analysis. If no ICD-10 code for one of the respective diseases was documented in the database, the case was handled as not having one of the described pre-existing conditions.

### Statistical analysis

Characteristics of the patients with the use of overactive bladder anticholinergic medications were compared to patients without. The ABS, ABS-U, and ABS-F were calculated for each case. Metric variables were tested for normal distribution using the Kolmogoroff-Smirnov test. We used medians and interquartile ranges (IQR) to describe the respective two groups. For categorical variables, we used absolute and relative frequencies. *p*-values were calculated using the Mann–Whitney *U* test for metric variables and the chi-squared test for categorical variables. Likewise, patients with the use of FRIDs were compared with patients without (Supplement 3).

We calculated unadjusted regression analyses along with two additional adjusted models for presenting with a fall to the ED always comparing the exposure to overactive bladder anticholinergic medications to no exposure.

Model 1 was adjusted for age and sex, and model 2 was adjusted for age, sex, number of prescribed FRIDs excluding overactive bladder anticholinergic medications, number of drugs (without FRIDs), ABS-U, and pre-existing conditions associated with falling (see above, all binary (yes vs. no)). An interaction term for the use of overactive bladder anticholinergic medications and the ABS-U was calculated using model 2.

The frequency of exposure and the applied dosages of single drug were analyzed, and for each single substance, the regression models were repeated as described above (models 1 and 2).

### Secondary analysis

For FRIDs, model 1 was calculated analogous to the model above. A model similar to model 2 was used adjusting for age, sex, number of drugs (without FRIDs), ABS-F and all pre-existing conditions associated with falling (see above) (model 3). Both models were repeated including the number of FRIDs per patient.

Results of logistic regression analyses are shown as odds ratios (OR) with corresponding 95% confidence intervals (CI). All statistical analyses were conducted using IBM^®^ SPSS^®^ Statistics (version 27).

## Results

In total, 2939 cases from the ADRED study were analyzed. Characteristics of the study population are displayed in Table 1. The majority of patients were not exposed to overactive bladder anticholinergic medications (97.8%, *n* = 2875). Despite that, fall as cause for the emergency visit was more frequent in patients with overactive bladder anticholinergic medications (17.2%, *n* = 11) compared to those without (5.6%, *n* = 160).

**Table 1:** Characteristics of study population according to exposure to overactive bladder anticholinergic medications or not

	**Missing cases**	**Without bladder medication, *n* = 2875**	**With bladder medication, *n* = 64**	***p*-value**
Age in years, *median (IQR)*	0	72 (57; 80)	80 (75; 86)	** < 0.001**
GFR in ml/min/1.73 m^2^, *median (IQR)*	793	64.64 (41.46; 86.73)	58.76 (36.51; 72.95)	**0.044**
Length of stay in days, *median (IQR)*	294	6 (3; 10)	7.5 (3; 14)	**0.023**
No. of admission diagnosis, *median (IQR)*	0	1 (1; 2)	2 (1; 3)	0.110
No. of prescribed drugs, *median (IQR)*	0	7 (3; 10)	10 (8; 14)	** < 0.001**
No. of ADR symptoms, *median (IQR)*	0	2 (1; 4)	2 (1; 3)	0.061
No. of pre-existing conditions, *median (IQR)*	262	5 (3; 7)	6 (3; 9)	0.132
No. of FRIDs, *median (IQR)*	0	1 (0; 3)	3 (2; 5)	** < 0.001**
No. of drugs taken (without FRIDs), *median (IQR)*	0	5 (2; 8)	6 (5; 10)	** < 0.001**
ABS, *median (IQR)*	0	1 (0; 2)	5 (4; 6)	** < 0.001**
ABS-U, *median (IQR)*	0	1 (0; 2)	2 (1; 3)	**0.001**
Sex, *n (%)*	0			0.735
Female		1421 (49.4)	33 (51.6)	
Male		1454 (50.6)	31 (48.4)	
Triage, *n (%)*	10			0.449
Red		152 (5.3)	2 (3.2)	
Orange		1162 (40.5)	23 (36.5)	
Yellow		1400 (48.8)	33 (52.4)	
Green		142 (5.0)	4 (6.3)	
Blue		10 (0.3)	1 (1.6)	
Seriousness of the ADR, *n (%)*	0			0.385
No serious damage		314 (10.9)	3 (4.7)	
Hospitalization required		2371 (82.5)	54 (84.4)	
Life-threatening damage		182 (6.3)	7 (10.9)	
Persistent damage		1 (0.0)	0 (0.0)	
Death		7 (0.2)	0 (0.0)	
Condition at discharge,* n (%)*	0			0.110
Recovered without damage		141 (4.9)	2 (3.1)	
Not yet recovered		260 (9.0)	11 (17.2)	
Improved condition		2196 (76.4)	42 (65.6)	
Permanent damage		16 (0.6)	1 (1.6)	
Death		110 (3.8)	2 (3.1)	
Treatment, *n (%)*	0			0.070
Inpatient		2590 (90.1)	62 (96.9)	
Outpatient		285 (9.9)	2 (3.1)	
Fall, *n (%)*	0	160 (5.6)	11 (17.2)	** < 0.001**

Bold emphasis: significance *p* < 0.05

*No.* number, *IQR* interquartile range, *ADR* adverse drug reaction, *FRIDS* fall-risk increasing drugs, *GFR* glomerular filtration rate, *ABS* anticholinergic burden score, *ABS-U* anticholinergic burden score without the anticholinergic burden of overactive bladder anticholinergic medications

Patients exposed to overactive bladder anticholinergic medications took in median 10 [8;14] drugs. FRID use in this population was as follows: Diuretics were taken by 57.8% (*n* = 34) of those, non-opioid analgesics by 29.7% (*n* = 19), antidepressants by 23.4% (*n* = 15), opioids by 21.9% (*n* = 14), antiepileptics by 20.3% (*n* = 13), antipsychotics, anti-Parkinson drugs, α-blocker for prostatic hyperplasia and sedatives/hypnotics/anxiolytics by 10.9% (*n* = 7) respectively, cardiac glycosides by 6.3% (*n* = 4), α-blocker as antihypertensive drugs by 3.7% (*n* = 3), and central acting antihypertensive drugs by 3.1% (*n* = 2). Use of certain FRIDs and fall predisposing diseases are pictured in Supplement 4.

**Fig. 1:**
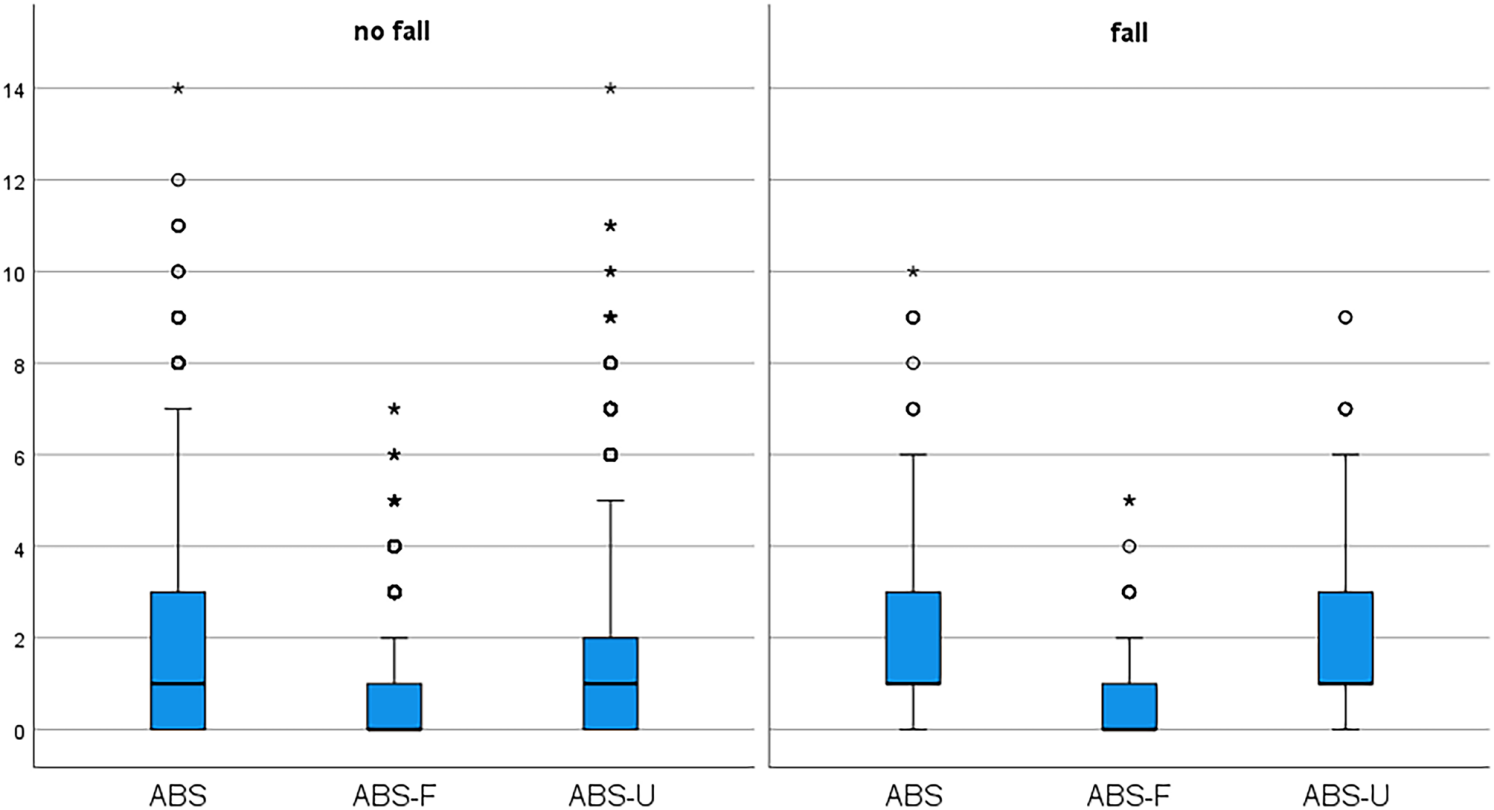
ABS, ABS-F, and ABS-U in patients with no fall versus fall. ABS: anticholinergic burden score; ABS-F: anticholinergic burden score excluding the anticholinergic properties of fall-risk increasing drugs; ABS-U: anticholinergic burden score excluding the anticholinergic properties of overactive bladder anticholinergic medications

The overall ABS was higher in patients with urologicals (ABS 5 [4;6] vs. 1 [0;2]). When excluding urologicals from the ABS, patients with urologicals still had a higher score (ABS-U 2 [1;3] vs. 1 [0;2]) (Fig. 1). Our study population took seven different overactive bladder anticholinergic medications, prescribed with various doses (Supplement 5).

Results from the regression analyses are displayed in Table 2. The unadjusted OR for overactive bladder anticholinergic medications was 3.46 [1.77–6.76] and decreased after adjusting for age and sex 2.77 [1.40–5.49]. Adjusting for further parameters showed an OR of 2.34 [1.14–4.82]. Adjusted model 2 for overactive bladder anticholinergic medications is shown completely in Table 3. Next to overactive bladder anticholinergic medications, age, female sex, the number of FRIDs, soft tissue diseases, abnormality of gait and mobility/ tendency to fall, and polyneuropathy were associated with falling in our sample. The ABS-U was not associated with falling in the regression model (OR 1.01 [0.90–1.12]).

**Table 2:** Regression analyses for the association of falls as adverse drug reaction with the exposure to overactive bladder anticholinergic medications in general, single overactive bladder anticholinergic medications, and fall-risk increasing drugs (FRIDs)

	Missing values, *n*	Unadjusted, OR [95% CI]	Model 1, OR [95% CI]	Model 2, OR [95% CI]	Model 3, OR [95% CI]
Bladder medication (yes)	262	**3.46 [1.77–6.76]**	**2.77 [1.40–5.49]**	**2.34 [1.14–4.82]**	**–**
Darifenacin (yes)	262	1.99 [0.25–15.98]	1.47 [0.18–12.14]	0.96 [0.10–9.61]	–
Oxybutynin (yes)	262	**9.67 [2.29–40.81]**	**9.34 [2.09–41.72]**	**9.20 [1.84–45.89]**	**–**
Solifenacin (yes)	262	3.98 [0.42–35.80]	3.09 [0.34–28.09]	1.46 [0.14–15.16]	–
Trospium (yes)	262	**3.25 [1.33–7.93]**	2.45 [0.99–6.06]	2.15 [0.83–5.53]	–
FRIDs (yes)	262	**2.89 [1.71–4.88]**	**2.30 [1.34–3.92]**	–	**2.30 [1.32–3.99]**
No. of FRIDs	262	**1.21 [1.12–1.31]**	**1.15 [1.06–1.25]**	**–**	**1.15 [1.05–1.26]**

Bold emphasis: significance *p* < 0.05

Model 1: adjusted for age and sex

Model 2: adjusted for age, sex, no. of FRIDs (excluding overactive bladder anticholinergic medications), no. of drugs taken (without FRIDs), pre-existing conditions (rheumatic diseases, diseases of the spine and back, soft tissue diseases, osteopathies and chondropathies, abnormality of gait and mobility/tendency to fall, dementia, Parkinson’s disease, multiple sclerosis, epilepsy, polyneuropathy, cerebral palsy and paralysis syndrome, affective, neurotic, stress and somatoform disorders, chronic pain, atherosclerosis of arteries of extremities and urinary incontinence), and anticholinergic burden score without the anticholinergic burden of overactive bladder anticholinergic medications

Model 3: adjusted for age, sex, no. of drugs taken (without FRIDs), pre-existing conditions (rheumatic diseases, diseases of the spine and back, soft tissue diseases, osteopathies and chondropathies, abnormality of gait and mobility/ tendency to fall, dementia, Parkinson’s disease, multiple sclerosis, epilepsy, polyneuropathy, cerebral palsy and paralysis syndrome, affective, neurotic, stress and somatoform disorders, chronic pain, atherosclerosis of arteries of extremities and urinary incontinence), and the anticholinergic burden score without the anticholinergic burden of FRIDs

**Table 3:** Full adjusted model 2 for the association of falls with exposure to overactive bladder anticholinergic medications

	OR [95% CI]
Bladder medication	**2.34 [1.14–4.82]**
Age	**1.03 [1.01–1.04]**
Sex (female)	**1.66 [1.16–2.36]**
No. of FRIDs (without urologicals)	**1.14 [1.02–1.27]**
No. of drugs (without FRIDs)	0.92 [0.87–0.98]
ABS-U (anticholinergic burden score without urologicals)	1.01 [0.90–1.12]
Rheumatic diseases	1.31 [0.71–2.43]
Diseases of the spine and back	1.32 [0.61–2.84]
Soft tissue diseases	**3.77 [1.39–10.22]**
Osteopathies and chondropathies	1.38 [0.67–2.83]
Abnormality of gait and mobility/tendency to fall	**2.96 [1.26–6.95]**
Dementia	1.67 [0.96–2.88]
Parkinson’s disease	1.35 [0.56–3.27]
Multiple sclerosis	1.97 [0.25–15.73]
Epilepsy	1.32 [0.51–3.43]
Polyneuropathy	**2.32 [1.17–4.60]**
Cerebral palsy and paralysis syndrome	1.26 [0.35–4.49]
Affective, neurotic, stress, and somatoform disorders	1.42 [0.84–2.41]
Chronic pain	1.01 [0.49–2.09]
Atherosclerosis of arteries of extremities	0.37 [0.13–1.04]
Urinary incontinence	1.12 [0.47–2.66]

Bold emphasis: significance *p* < 0.05

*FRIDS* fall-risk increasing drugs, *ABS-U* anticholinergic burden score without the anticholinergic burden of overactive bladder anticholinergic medications

There was no significant interaction term between the use of overactive bladder anticholinergic medications and ABS-U (*p* = 0.26).

We calculated regression analyses for each of the seven overactive bladder anticholinergic medications individually. Both oxybutynin (OR 9.67 [2.29–40.81]) and trospium (OR 3.25 [1.33–7.93]) were significantly associated with higher chances for falls in unadjusted analyses, but with large confidence intervals due to little statistical power. Only oxybutynin showed significant results in model 1 (OR 9.34 [2.09–41.72]) as well as model 2 (OR 9.20 [1.84–45.89]), while trospium did not.

### Secondary analysis

Also exposure to FRIDs was associated with falls in logistic regression analyses. This was true for the use of FRIDs as well as for an increase of odds with higher number of FRIDs (Table 2).

## Discussion

This analysis of falls that led to ED visits, showed that not only FRIDs in general, but more specifically overactive bladder anticholinergic medications are associated with an increased risk for falls. While the anticholinergic burden was higher in patients with FRIDs and overactive bladder anticholinergic medications, these drugs seemed to be associated with falls irrespective of the cumulative anticholinergic burden caused by the concomitant multi-medication.

With an adjusted OR of 2.34, the risk for presenting with a fall to the ED is doubled when taking an overactive bladder anticholinergic medication compared to controls. Due to anticholinergic activity, this association is a reasonable assumption for fall genesis in older adults. The cumulative anticholinergic activity respecting other anticholinergic drugs such as specific antidepressants seemed to have less impact on fall-risks in our analysis. We could not observe an interaction of anticholinergic activity with the use of overactive bladder anticholinergic medications. Thus, the fall-risk increasing effect may be connected to overactive bladder anticholinergic medications irrespective of the anticholinergic activity deriving from the other drugs taken concomitantly.

Discriminating between effects of drugs and effects of the underlining disease is always a challenge (i.e., confounding by indication). It has previously been shown that overactive bladder (OAB) itself is associated with an increased risk of falls [24]. This might be related to the urinary urgency associated with the OAB that causes sudden movements which could be problematic in older adults with gait impairment. While patients with OAB seem to be at higher risk for falls, some evidence might point towards a reduced risk for falls when treated [25].

In contrast, evidence for falls in patients with OAB treated with anticholinergic drugs can likewise be found [26]. Thus, it remains unclear whether the OAB, or the anticholinergic therapy is the reason for the increased risk for falls or a combination of both.

Our finding of an increased risk for falls with overactive bladder anticholinergic medications is in line with a consensus on FRIDs that refers fall-risks to the anticholinergic activity of drugs [9]. Regarding individual overactive bladder anticholinergic medications, differences between single drugs should be expected, in line with different anticholinergic burdens per drug. But our study population was too small for final conclusions. One might differentiate between selective (solifenacin, darifenacin) and non-selective (oxybutynin, tolterodine, trospium, and fesoterodine) anticholinergics supported by a study indicating a significant risk for non-selective anticholinergic in patients [27, 28]. Also, the time of use might be of importance for the individual fall-risk, as some drugs could be expected to increase the fall-risk with continuous use [29]. The anticholinergic burden of overactive bladder anticholinergic medications is associated with cognitive decline and could be connected with different altered risks for falls in older adults per single drugs [30]. Therefore, the anticholinergic capacity might outweigh the benefit of treating OAB pharmacologically in older adults in some cases.

The high observed prevalence of pre-existing conditions analyzed predisposing for falls might underline a multifactorial pathogenesis of falls in older adults. However, while also other non-pharmacological treatment options for OAB exist [31], the indication for a pharmacological treatment in older adults should be decided with caution.

An increased risk of falls with drug exposure might as well be associated with the intake of a multitude of drugs summing up to an increased risk with different drug-drug interactions. In our analyses, we respected this by adjusting for FRIDs, use of other drugs, and the cumulative anticholinergic burden. As this is a clear strength of our study, one need to admit that a distinct classification of anticholinergic drugs is lacking. Therefore, we combined different scoring systems on anticholinergic drugs [12].

Interestingly, while the number of FRIDs was associated with higher fall risks in fully adjusted models, the solely number of drugs excluding FRIDs was not. This might underline the importance of a summative effect of drugs associated with a fall-risk than the solely multi-medication.

We need to point out that this was a cohort of ADR cases and therefore comparisons derived from comparing with other ADR cases. However, the population covers a large picture of chief complaints for presenting to the ED and presents an older, multi-medicated population which is in line with other studies [11]. Besides, all ADR cases analyzed were assessed concerning causality using a standardized assessment, which is a clear strength of our study.

Our analysis has some limitations. Firstly, we calculated the ABS based solely on the fact whether a patient took an anticholinergic drug or not but did not differentiate between application forms. However, in our study population, most of the drugs act systemically. Secondly, due to the rather small sample size of patients with overactive bladder anticholinergic medications, we did not take individual dosages into account. Therefore, further investigation is needed on the effect of different dosages and their influence on falls. Other limitations lie in the study design as this was an observational study, data quality and completeness can differ from case to case and study center to study center. Also, the depth of documentation could differ from case to case with, e.g., sometimes serious cases that could not be interrogated well for their drug use and past medical history due to urgency of the symptoms. Thus, for a better harmonization of data, regular telephone conferences were conducted during enrollment between the study centers.

## Conclusion

In conclusion, we showed that overactive bladder anticholinergic medications are associated with an increased risk for falls leading to presentations to the ED. In addition, patients with FRIDs also have an increased risk for falls. While many drugs show anticholinergic properties, the fall-risk associated with overactive bladder anticholinergic medication exposure seems increase despite anticholinergic load of the concomitant medication.

Although falls occur multifactorial in older adults and confounding by indication cannot be ruled out, the indication for a drug treatment should be decided with caution. Other, non-pharmacological treatment options such as behavioral therapy for OAB should be considered as well.

## Supplementary Information

Below is the link to the electronic supplementary material.

Supplementary file1 (DOCX 56 KB)

## Data Availability

The datasets generated during the current study are available from the corresponding author on reasonable request.

## References

[1] Kannus P, Parkkari J, Koskinen S, Niemi S, Palvanen M, Järvinen M, Vuori I (1999). Fall-induced injuries and deaths among older adults. JAMA.

[2] Berry SD, Miller RR (2008). Falls: epidemiology, pathophysiology, and relationship to fracture. Curr Osteoporos Rep.

[3] Shankar KN, Liu SW, Ganz DA (2017). Trends and characteristics of emergency department visits for fall-related injuries in older adults, 2003–2010. West J Emerg Med.

[4] Phelan EA, Ritchey K (2018) Fall prevention in community-dwelling older adults. Ann Intern Med 169 (11):Itc81–itc96. 10.7326/aitc201812040 10.7326/AITC201812040 30508457

[5] Hwang S, Jun K, Ah YM, Han E, Chung JE, Lee JY (2019) Impact of anticholinergic burden on emergency department visits among older adults in Korea: a national population cohort study. Arch Gerontol Geriatr 85:103912. 10.1016/j.archger.2019.103912 10.1016/j.archger.2019.103912 31386937

[6] Green AR, Reifler LM, Bayliss EA, Weffald LA, Boyd CM (2019). Drugs contributing to anticholinergic burden and risk of fall or fall-related injury among older adults with mild cognitive impairment, dementia and multiple chronic conditions: a retrospective cohort study. Drugs Aging.

[7] Seppala LJ, van de Glind EMM, Daams JG, Ploegmakers KJ, de Vries M, Wermelink A, van der Velde N, Task E, Finish Group on Fall-Risk-Increasing D (2018) Fall-risk-increasing drugs: a systematic review and meta-analysis: III. Others. J Am Med Dir Assoc 19(4):e371–372, e378. 10.1016/j.jamda.2017.12.099 10.1016/j.jamda.2017.12.099 29402646

[8] Stingl JC, Just KS, Schurig M, Böhme M, Steffens M, Schwab M, Seufferlein T, Dormann H (2020). Prevalence of psychotropic drugs in cases of severe adverse drug reactions leading to unplanned emergency visits in general hospitals. Pharmacopsychiatry.

[9] Seppala LJ, Petrovic M, Ryg J, Bahat G, Topinkova E, Szczerbinska K, van der Cammen TJM, Hartikainen S, Ilhan B, Landi F, Morrissey Y, Mair A, Gutierrez-Valencia M, Emmelot-Vonk MH, Mora MAC, Denkinger M, Crome P, Jackson SHD, Correa-Perez A, Knol W, Soulis G, Gudmundsson A, Ziere G, Wehling M, O'Mahony D, Cherubini A, van der Velde N (2020). STOPPFall (Screening Tool of Older Persons Prescriptions in older adults with high fall risk): a Delphi study by the EuGMS Task and Finish Group on Fall-Risk-Increasing Drugs. Age Ageing.

[10] American Geriatrics Society (2019). Updated AGS Beers Criteria® for potentially inappropriate medication use in older adults. J Am Geriatr Soc.

[11] Schurig AM, Bohme M, Just KS, Scholl C, Dormann H, Plank-Kiegele B, Seufferlein T, Graff I, Schwab M, Stingl JC (2018). Adverse drug reactions (ADR) and emergencies. Deutsches Arzteblatt international.

[12] Salahudeen MS, Duffull SB, Nishtala PS (2015). Anticholinergic burden quantified by anticholinergic risk scales and adverse outcomes in older people: a systematic review. BMC Geriatr.

[13] Ikutomo H, Nagai K, Tagomori K, Miura N, Nakagawa N, Masuhara K (2019). Incidence and risk factors for falls in women with end-stage hip osteoarthritis. J Geriatr Phys Ther.

[14] Stanmore EK, Oldham J, Skelton DA, O'Neill T, Pilling M, Campbell AJ, Todd C (2013). Risk factors for falls in adults with rheumatoid arthritis: a prospective study. Arthritis Care Res (Hoboken).

[15] Dursun N, Sarkaya S, Ozdolap S, Dursun E, Zateri C, Altan L, Birtane M, Akgun K, Revzani A, Aktas I, Tastekin N, Celiker R (2015). Risk of falls in patients with ankylosing spondylitis. J Clin Rheumatol.

[16] Moreland JD, Richardson JA, Goldsmith CH, Clase CM (2004). Muscle weakness and falls in older adults: a systematic review and meta-analysis. J Am Geriatr Soc.

[17] Yeung SSY, Reijnierse EM, Pham VK, Trappenburg MC, Lim WK, Meskers CGM, Maier AB (2019). Sarcopenia and its association with falls and fractures in older adults: a systematic review and meta-analysis. J Cachexia Sarcopenia Muscle.

[18] Salzman B (2010). Gait and balance disorders in older adults. Am Fam Physician.

[19] Allan LM, Ballard CG, Rowan EN, Kenny RA (2009) Incidence and prediction of falls in dementia: a prospective study in older people. PLoS One 4 (5):e5521. 10.1371/journal.pone.0005521 10.1371/journal.pone.0005521 PMC2677107 19436724

[20] Bloem BR, Grimbergen YA, Cramer M, Willemsen M, Zwinderman AH (2001). Prospective assessment of falls in Parkinson's disease. J Neurol.

[21] Sosnoff JJ, Socie MJ, Boes MK, Sandroff BM, Pula JH, Suh Y, Weikert M, Balantrapu S, Morrison S, Motl RW (2011) Mobility, balance and falls in persons with multiple sclerosis. PLoS One 6 (11):e28021. 10.1371/journal.pone.0028021 10.1371/journal.pone.0028021 PMC3222674 22132196

[22] Wirrell EC (2006). Epilepsy-related injuries. Epilepsia.

[23] Hanewinckel R, Drenthen J, Verlinden VJA, Darweesh SKL, van der Geest JN, Hofman A, van Doorn PA, Ikram MA (2017). Polyneuropathy relates to impairment in daily activities, worse gait, and fall-related injuries. Neurology.

[24] Szabo SM, Gooch KL, Walker DR, Johnston KM, Wagg AS (2018). The association between overactive bladder and falls and fractures: a systematic review. Adv Ther.

[25] Jayadevappa R, Chhatre S, Newman DK, Schwartz JS, Wein AJ (2018). Association between overactive bladder treatment and falls among older adults. Neurourol Urodyn.

[26] Yehoshua A, Chancellor M, Vasavada S, Malone DC, Armstrong EP, Joshi M, Campbell K, Pulicharam R (2016). Health resource utilization and cost for patients with incontinent overactive bladder treated with anticholinergics. J Manag Care Spec Pharm.

[27] Kachru N, Holmes HM, Johnson ML, Chen H, Aparasu RR (2021). Comparative risk of adverse outcomes associated with nonselective and selective antimuscarinic medications in older adults with dementia and overactive bladder. Int J Geriatr Psychiatry.

[28] Gomes T, Juurlink DN, Ho JM, Schneeweiss S, Mamdani MM (2011). Risk of serious falls associated with oxybutynin and tolterodine: a population based study. J Urol.

[29] Welk B, Etaby K, McArthur E, Chou Q (2022). The risk of delirium and falls or fractures with the use of overactive bladder anticholinergic medications. Neurourol Urodyn.

[30] Wagg A, Verdejo C, Molander U (2010). Review of cognitive impairment with antimuscarinic agents in elderly patients with overactive bladder. Int J Clin Pract.

[31] Gormley EA, Lightner DJ, Burgio KL, Chai TC, Clemens JQ, Culkin DJ, Das AK, Foster HE, Scarpero HM, Tessier CD, Vasavada SP (2012). Diagnosis and treatment of overactive bladder (non-neurogenic) in adults: AUA/SUFU guideline. J Urol.

