# Supplementary material for: Use of overactive bladder anticholinergic medications associated with falls leading to emergency department visits: results from the ADRED study
Source: Eur J Clin Pharmacol. 2023 Jun 29;79(9):1185–93. doi: 10.1007/s00228-023-03530-3 (PMC10427532; doi:10.1007/s00228-023-03530-3)
Supplement: Supplementary file 1 — Supplementary file1 (DOCX 56 KB) [file 228_2023_3530_MOESM1_ESM.docx]

# **Use of overactive bladder anticholinergic medications associated with falls leading to emergency department visits – results from the ADRED study**

Katja S Just*^1^ (ORCID: 0000-0002-6782-8078), Karen A Schultze*^1^, Harald Dormann^2^, Thomas Seufferlein^3^, Ingo Gräff^4^, Catharina Scholl^5^, Matthias Schwab^6,7,8^, Julia C Stingl^1^

1 Institute of Clinical Pharmacology, University Hospital RWTH Aachen, Aachen, Germany

2 Central Emergency Department, Hospital Fürth, Fürth, Germany

3 Internal Medicine Emergency Department, Ulm University Medical Centre, Ulm, Germany

4 Interdisciplinary Emergency Department (INZ), University Hospital of Bonn, Bonn, Germany

5 Research Department, Federal Institute for Drugs and Medical Devices, Bonn, Germany

6 Dr. Margarete Fischer-Bosch-Institute of Clinical Pharmacology, Stuttgart, Germany

7 Department of Clinical Pharmacology, University of Tuebingen, Tuebingen, Germany

8 Department of Pharmacy and Biochemistry, University of Tuebingen, Tuebingen, Germany

*Contributed equally

Correspondence

PD Dr. med. Katja S Just

Institute of Clinical Pharmacology

University Hospital of

RWTH Aachen

Wendlingweg 2

D-52074 Aachen

kjust@ukaachen.de

## **Supplement**

### Supplement 1: included drug groups assessed as fall-risk increasing defined by ATC codes

N06A Antidepressants

N03A Antiepileptics

N05A Antipsychotics

C03 Diuretics

CO2L, C07B, C07C, C07D, C08G, C09BA combined diuretics

N02A Opioids

N05B, N05C Sedatives, Hypnotics, Anxiolytics

C02CA a-blocker as antihypertensive drugs

G04CA a-Blocker for prostatic hyperplasia

G04BD Overactive bladder anticholinergic medications

C02D Vasodilators for cardiovascular diseases

C02A Central acting antihypertensive drugs

C01A Cardiac glycosides

M01A, N02B Non-opioid analgesics

N04 Anti-Parkinson drugs

Benzodiazepines and antihistamines were not included individually, but in the group of sedatives, hypnotics, and anxiolytics for low occurrence in the dataset [1]. In addition, cardiac glycosides [2], non-opioid analgesics [1, 3, 4], and anti-Parkinson [3] drugs were included.

References

1. Woolcott, J.C., et al., *Meta-analysis of the impact of 9 medication classes on falls in elderly persons.* Arch Intern Med, 2009. **169**(21): p. 1952-60.

2. de Vries, M., et al., *Fall-Risk-Increasing Drugs: A Systematic Review and Meta-Analysis: I. Cardiovascular Drugs.* J Am Med Dir Assoc, 2018. **19**(4): p. 371 e1-371 e9.

3. Seppala, L.J., et al., *Fall-Risk-Increasing Drugs: A Systematic Review and Meta-analysis: III. Others.* J Am Med Dir Assoc, 2018. **19**(4): p. 372 e1-372 e8.

4. Zia, A., S.B. Kamaruzzaman, and M.P. Tan, *The consumption of two or more fall risk-increasing drugs rather than polypharmacy is associated with falls.* Geriatr Gerontol Int, 2017. **17**(3): p. 463-470.

### Supplement 2: included anticholinergic drugs and final anticholinergic burden score used in this study

| Drugs | Reported score by Salahudeen et al. (no. of studies supporting score) | Final Score |
| --- | --- | --- |
| Alprazolam | 3 (1), 1 (4)^1^ | 1 |
| Amantadine | 2 (2), 1 (2)^2^ | 2 |
| Amitriptyline | 3 | 3 |
| Ampicillin | 1 | 1 |
| Aripiprazol | 1 | 1 |
| Atenolol | 1 | 1 |
| Atropine | 3 | 3 |
| Azathioprine | 1 | 1 |
| Baclofen | 2 | 2 |
| Benazepril | 1 | 1 |
| Betaxolol | 1 | 1 |
| Bisacodyl | 1 | 1 |
| Bupropion | 1 | 1 |
| Captopril | 1 | 1 |
| Carbamazepine | 2 (2), 1 (1) | 2 |
| Carbidopa | 1 | 1 |
| Celecoxib | 1 | 1 |
| Cetirizine/ Levocetirizine | 2 (3), 1 (1) | 2 |
| Chlorphenamine | 3 | 3 |
| Chlorthalidone | 1 | 1 |
| Cimetidine | 2 (2), 1 (1) | 2 |
| Citalopram/ Escitalopram | 1 | 1 |
| Clindamycin | 1 | 1 |
| Clomipramine | 3 | 3 |
| Clonazepam | 1 | 1 |
| Clozapine | 3 (3), 2 (1) | 3 |
| Codeine | 2 (1), 1 (4) | 1 |
| Colchicine | 3 (1), 1 (1) | 3 |
| Cortisone/ Hydrocortisone/ Fludrocortisone | 1 | 1 |
| Cyclosporine | 1 | 1 |
| Darifenacin | 3 | 3 |
| Desloratidine | 1 | 1 |
| Dexamethasone | 1 | 1 |
| Dextromethorphan | 1 | 1 |
| Diazepam | 1 | 1 |
| Digitoxin | 1 | 1 |
| Digoxin | 3 (1), 1 (4) | 1 |
| Diltiazem | 1 | 1 |
| Dimenhydrinate | 3 | 3 |
| Diphenhydramine | 3 | 3 |
| Domperidone | 1 | 1 |
| Doxepin | 3 | 3 |
| Doxylamine | 3 | 3 |
| Entacapone | 1 | 1 |
| Famotidine | 1 | 1 |
| Fentanyl | 1 | 1 |
| Fesoterodine | 3 | 3 |
| Fexofenadine | 2 | 2 |
| Fluoxetine | 1 | 1 |
| Fluphenazine | 3 (2), 1 (1) | 3 |
| Fluticasone-salmeterol | 1 | 1 |
| Fluvoxamine | 1 | 1 |
| Furosemide | 3 (1), 1 (4) | 1 |
| Gentamicin | 1 | 1 |
| Guaifenesin | 1 | 1 |
| Haloperidol | 2 (1), 1 (2) | 1 |
| Hydralazine (Dihydralazine) | 1 | 1 |
| Hydrocortisone | 1 | 1 |
| Hydroxyzine | 3 | 3 |
| Ipratropium (including inhalation) | 3 | 3 |
| Isosorbide (including Mono-/Dinitrate) | 1 | 1 |
| Levomepromazine | 3 | 3 |
| Lithium | 1 | 1 |
| Loperamide | 2 (1), 1 (4) | 1 |
| Loratadine | 2 (1), 1 (3) | 1 |
| Lorazepam | 1 | 1 |
| Maprotiline | 3 | 3 |
| Metformin | 1 | 1 |
| Methadone (including Levomethadone) | 2 | 2 |
| Methocarbamol | 1 | 1 |
| Methotrexate | 1 | 1 |
| Metoprolol | 1 | 1 |
| Midazolam | 1 | 1 |
| Mirtazapine | 1 | 1 |
| Morphine | 1 | 1 |
| Naratriptan | 1 | 1 |
| Nifedipine | 1 | 1 |
| Olanzapine | 3 (1), 2 (2), 1 (2) | 2 |
| Oxazepam | 1 | 1 |
| Oxcarbazepine | 2 | 2 |
| Oxybutynin | 3 (5), 2 (1) | 3 |
| Oxycodone | 1 | 1 |
| Paliperidone | 1 | 1 |
| Paroxetine | 3 (1), 2 (3), 1 (2) | 2 |
| Phenobarbital | 1 | 1 |
| Piperacillin | 1 | 1 |
| Pramipexole | 1 | 1 |
| Prednisolone (including. Methylprednisolone) | 1 | 1 |
| Prednisone | 1 | 1 |
| Promethazine | 3 | 3 |
| Propiverine | 3 (2), 2 (1) | 3 |
| Pseudoephedrine | 2 | 2 |
| Quetiapine | 3 (1), 2 (1), 2 (2) | 1 |
| Quinidine | 1 | 1 |
| Ranitidine | 2 (2), 1 (4) | 1 |
| Reglan | 3 | 3 |
| Risperidone | 1 | 1 |
| Scopolamine(hyoscine) (Butylscopolamin) | 3 | 3 |
| Sertraline | 1 | 1 |
| Solifenacin | 3 | 3 |
| Sumatriptan | 1 | 1 |
| Temazepam | 1 | 1 |
| Theophylline | 2 (2), 1 (3) | 1 |
| Tizanidine | 3 | 3 |
| Tolterodine | 3 (5), 2 (1) | 3 |
| Tramadol | 2 (2), 1 (1) | 2 |
| Trazodone | 1 | 1 |
| Triamcinolone | 1 | 1 |
| Triamterene | 1 | 1 |
| Trimipramine | 3 | 3 |
| Trospium | 3 | 3 |
| Valproic acid | 1 | 1 |
| Vancomycin | 1 | 1 |
| Venlafaxine | 1 | 1 |

In the medication list of drugs used in the ADRED study, 73 drugs were not found in the 195 drugs listed by Salahudeen et al.

107 drugs were used to calculate the anticholinergic burden score for each patient in Model 2 (Urologicals) and 49 drugs for the anticholinergic burden score for Model 3 (FRIDs).

For some drugs more than one anticholinergic burden score was given.

1: We used the score with more evidence based on the viewed literature.

2: We used the higher score if there was an equal number of literature sources.

Three drugs were included twice in the anticholinergic burden scale with different names (Levomepromazine/Methotrimeprazine, Metoclopramide/Reglan, Robitussin/Dextromethorphan) leading to different anticholinergic scores. For this analysis, we always used the higher score given by Salahudeen et al.

To score the anticholinergic risk, if possible, comparable drugs were used that were found in the list. This was the case for example for Escitalopram, listed was Citalopram, Levocetirizine, listed was Cetirizine, Methylprednisolone, listed was Prednisolone.

For three drugs, other derivates of those drugs were taken in ADRED and included under the same variable. This was the case for Cortisone, also included were Hydrocortisone and Fludrocortisone, Isosorbide, also included were Isosorbide mono- and dinitrate, and Methadone, also included was Levomethadone.

For four drugs only one drug version was included, Hydralazine, only Dihydralazine, Promazine, only Levomepromazine, Quinidine, only Quinine, and Scopolamine, only Butylscopolamine.

## Supplement 3: Characteristics of study population according to taking FRIDs or not

|  | **missing cases** | **without FRIDs, n=783** | **with FRIDs, n=2156** | **p-Value** |
| --- | --- | --- | --- | --- |
| Age in years, *Median (IQR)* | 0 | 61 (46; 75) | 75 (63; 82) | **<0.001** |
| BMI, *Median (IQR)* | 1939 | 25.2 (21.5; 28.6) | 25.8 (22.9; 29.6) | **0.001** |
| GFR in ml/min/1.73 m², *Median (IQR)* | 793 | 80.13 (58.10; 97.20) | 58.07 (37.01; 82.03) | **<0.001** |
| Length of stay in days, *Median (IQR)* | 294 | 5 (2; 8) | 6 (3; 10) | **<0.001** |
| No. of admission diagnosis, *Median (IQR)* | 0 | 1 (1; 2) | 1 (1; 2) | **<0.001** |
| No. of prescribed drugs, *Median (IQR)* | 0 | 3 (1; 5) | 8 (6; 11) | **<0.001** |
| No. of ADR symptoms, *Median (IQR)* | 0 | 2 (1; 3) | 2 (1; 4) | 0.914 |
| No. of pre-existing conditions, *Median (IQR)* | 262 | 3 (2; 5) | 5 (3; 8) | **<0.001** |
| No. of drugs taken (without FRIDs), *Median (IQR)* | 0 | 3 (1; 5) | 6 (4; 8) | **<0.001** |
| ABS, *Median (IQR)* | 0 | 0 (0; 1) | 2 (1; 3) | **<0.001** |
| ABS-F, *Median*  *(IQR)* | 0 | 0 (0; 1) | 0 (0; 1) | **<0.001** |
| Sex, *n (%)* | 0 |  |  | 0.524 |
| female |  | 395 (50.4) | 1059 (49.1) |  |
| male |  | 388 (49.6) | 1097 (50.9) |  |
| Triage, *n (%)* | 10 |  |  | **0.016** |
| red |  | 32 (4.1) | 122 (5.7) |  |
| orange |  | 303 (38.3) | 882 (41.1) |  |
| yellow |  | 416 (53.3) | 1017 (47.3) |  |
| green |  | 28 (3.6) | 118 (5.5) |  |
| blue |  | 2 (0.3) | 9 (0.4) |  |
| Seriousness of the ADR, *n (%)* | 0 |  |  | **<0.001** |
| no serious damage |  | 171 (21.8) | 146 (6.8) |  |
| hospitalization required |  | 566 (72.3) | 1859 (86.2) |  |
| life-threatening damage |  | 43 (5.5) | 146 (6.8) |  |
| persistent damage |  | 0 (0.0) | 1 (0.0) |  |
| death |  | 3 (0.4) | 4 (0.2) |  |
| Condition at discharge, *n (%)* | 0 |  |  | 0.575 |
| recovered without damage |  | 44 (5.6) | 99 (4.6) |  |
| not yet recovered |  | 68 (8.7) | 203 (9.4) |  |
| improved condition |  | 598 (76.4) | 1640 (76.1) |  |
| permanent damage |  | 6 (0.8) | 11 (0.5) |  |
| death |  | 24 (3.1) | 88 (4.1) |  |
| Treatment, *n (%)* | 0 |  |  | **<0.001** |
| inpatient |  | 626 (79.9) | 2026 (94.0) |  |
| outpatient |  | 157 (20.1) | 130 (6.0) |  |
| Falls, *n (%)* | 0 | 24 (3.1) | 147 (6.8) | **<0.001** |
| Pre-existing conditions, *n (%)* | 262 | 123 (19.5) | 906 (44.3) | **<0.001** |
| Rheumatic diseases | 262 | 23 (3.6) | 151 (7.4) | **0.001** |
| Rheumatoid arthritis | 262 | 9 (1.4) | 58 (2.8) | **0.048** |
| Arthrosis | 262 | 2 (0.3) | 41 (2.0) | **0.003** |
| Gout | 262 | 2 (0.3) | 19 (0.9) | 0.128 |
| Connective tissue diseases | 262 | 9 (1.4) | 38 (1.9) | 0.471 |
| Diseases of the spine and back | 262 | 7 (1.1) | 82 (4.0) | **<0.001** |
| Ankylosing spondylitis | 262 | 2 (0.3) | 2 (0.1) | 0.213 |
| Soft tissue diseases | 262 | 7 (1.1) | 21 (1.0) | 0.858 |
| Muscular diseases | 262 | 3 (0.5) | 11 (0.5) | 0.850 |
| Sarcopenia | 262 | 3 (0.5) | 10 (0.5) | 0.966 |
| Osteopathies and Chondropathies | 262 | 17 (2.7) | 78 (3.8) | 0.184 |
| Osteoporosis | 262 | 14 (2.2) | 68 (3.3) | 0.159 |
| Abnormalities of gait and mobility/ tendency to fall | 262 | 4 (0.6) | 36 (1.8) | **0.042** |
| Dementia | 262 | 16 (2.5) | 131 (6.4) | **<0.001** |
| Dementia in Alzheimer´s diseases | 262 | 1 (0.2) | 1 (0.0) | 0.378 |
| Vascular dementia | 262 | 2 (0.3) | 28 (1.4) | **0.028** |
| Dementia in other diseases classified elsewhere | 262 | 0 (0.0) | 2 (0.1) | 0.432 |
| Unspecified dementia | 262 | 9 (1.4) | 85 (4.2) | **0.001** |
| Parkinson´s disease | 262 | 2 (0.3) | 51 (2.5) | **0.001** |
| Multiple Sclerosis | 262 | 10 (1.6) | 7 (0.3) | **0.001** |
| Epilepsy | 262 | 0 (0.0) | 58 (2.8) | **<0.001** |
| Polyneuropathy | 262 | 8 (1.3) | 83 (4.1) | **0.001** |
| Cerebral Palsy and paralysis syndrome | 262 | 2 (0.3) | 32 (1.6) | **0.014** |
| Affective, neurotic, stress, and somatoform disorders | 262 | 14 (2.2) | 208 (10.2) | **<0.001** |
| Chronic pain | 262 | 4 (0.6) | 100 (4.9) | **<0.001** |
| Atherosclerosis of arteries of extremities | 262 | 25 (4.0) | 147 (7.2) | **0.004** |
| Urinary incontinence | 262 | 5 (0.8) | 54 (2.6) | **0.006** |

No.: number, IQR: interquartile range, ADR: adverse drug reaction, FRIDS: fall-risk increasing drugs, GFR: glomerular filtration rate, ABS: anticholinergic burden score, ABS-F: anticholinergic burden score without the anticholinergic burden of FRIDs

## Supplement 4: Characteristics of study population according to exposure to overactive bladder anticholinergic medications or not

|  | **missing cases** | **without bladder medication, n=2875** | **with bladder medication, n=64** | **p-Value** |
| --- | --- | --- | --- | --- |
| FRIDs taken, *n (%)* | 0 | 2092 (72.8) | 64 (100.0) | **<0.001** |
| Antidepressants | 0 | 482 (16.8) | 15 (23.4) | 0.159 |
| Antiepileptics | 0 | 303 (10.5) | 13 (20.3) | **0.013** |
| Antipsychotics | 0 | 187 (6.5) | 7 (10.9) | 0.158 |
| Diuretics | 0 | 1244 (43.4) | 37 (57.8) | **0.020** |
| Opioids | 0 | 438 (15.2) | 14 (21.9) | 0.145 |
| Sedatives, Hypnotics, Anxiolytics | 0 | 232 (8.1) | 7 (10.9) | 0.406 |
| a-blocker as antihypertensive drugs | 0 | 74 (2.6) | 3 (4.7) | 0.295 |
| a-blocker for prostatic hyperplasia | 0 | 225 (7.8) | 7 (10.9) | 0.361 |
| Vasodilators for cardiovascular diseases | 0 | 13 (0.5) | 0 (0.0) | 0.590 |
| Central acting antihypertensive drugs | 0 | 70 (2.4) | 2 (3.1) | 0.724 |
| Cardiac glycosides | 0 | 175 (6.1) | 4 (6.3) | 0.957 |
| Non-opioid analgesics | 0 | 809 (28.1) | 19 (29.7) | 0.785 |
| Anti-Parkinson drugs | 0 | 101 (3.5) | 7 (10.9) | **0.002** |
| Pre-existing conditions, *n (%)* | 262 | 988 (37.8) | 41 (64.1) | **<0.001** |
| Rheumatic diseases | 262 | 168 (6.4) | 6 (9.4) | 0.345 |
| Rheumatoid arthritis | 262 | 65 (2.5) | 2 (3.1) | 0.747 |
| Arthrosis | 262 | 41 (1.6) | 2 (3.1) | 0.328 |
| Gout | 262 | 20 (0.8) | 1 (1.6) | 0.475 |
| Connective tissue diseases | 262 | 46 (1.8) | 1 (1.6) | 0.905 |
| Diseases of the spine and back | 262 | 87 (3.3) | 2 (3.1) | 0.928 |
| Ankylosing spondylitis | 262 | 4 (0.2) | 0 (0.0) | 0.754 |
| Soft tissue diseases | 262 | 24 (0.9) | 4 (6.3) | **<0.001** |
| Muscular diseases | 262 | 10 (0.4) | 4 (6.3) | **<0.001** |
| Sarcopenia | 262 | 10 (0.4) | 3 (4.7) | **<0.001** |
| Osteopathies and Chondropathies | 262 | 92 (3.5) | 3 (4.7) | 0.618 |
| Osteoporosis | 262 | 79 (3.0) | 3 (4.7) | 0.445 |
| Abnormalities of gait and mobility/ tendency to fall | 262 | 37 (1.4) | 3 (4.7) | **0.033** |
| Dementia | 262 | 141 (5.4) | 6 (9.4) | 0.167 |
| Dementia in Alzheimer´s diseases | 262 | 1 (0.0) | 1 (1.6) | **<0.001** |
| Vascular dementia | 262 | 28 (1.1) | 2 (3.1) | 0.123 |
| Dementia in other diseases classified elsewhere | 262 | 2 (0.1) | 0 (0.0) | 0.825 |
| Unspecified dementia | 262 | 91 (3.5) | 3 (4.7) | 0.605 |
| Parkinson´s disease | 262 | 50 (1.9) | 3 (4.7) | 0.116 |
| Multiple Sclerosis | 262 | 16 (0.6) | 1 (1.6) | 0.344 |
| Epilepsy | 262 | 55 (2.1) | 3 (4.7) | 0.161 |
| Polyneuropathy | 262 | 87 (3.3) | 4 (6.3) | 0.203 |
| Cerebral Palsy and paralysis syndrome | 262 | 32 (1.2) | 2 (3.1) | 0.180 |
| Affective, neurotic, stress, and somatoform disorders | 262 | 214 (8.2) | 8 (12.5) | 0.217 |
| Chronic pain | 262 | 99 (3.8) | 5 (7.8) | 0.100 |
| Atherosclerosis of arteries of extremities | 262 | 169 (6.5) | 3 (4.7) | 0.566 |
| Urinary incontinence | 262 | 52 (2.0) | 7 (10.9) | **<0.001** |

Supplement 5: Overactive bladder anticholinergic medication use and doses

| Drug | Total No. of cases, n | No. of cases per dose, n | Dose (mg) |
| --- | --- | --- | --- |
| Darifenacin | 9 | 9 | 7.5 |
| Fesoterodine | 2 | 2 | 8.0 |
| Oxybutynin | 9 |  |  |
|  |  | 1 | 3.9 |
|  |  | 1 | 20.0 |
|  |  | 1 | 15.0 |
|  |  | 1 | 10.0 |
|  |  | 3 | 5.0 |
|  |  | 1 | 2.5 |
|  |  | 1 | unknown |
| Propiverine | 3 |  |  |
|  |  | 1 | 15.0 |
|  |  | 1 | 30.0 |
|  |  | 1 | unknown |
| Solifenacin | 5 |  |  |
|  |  | 3 | 5.0 |
|  |  | 2 | 10.0 |
| Tolterodine | 3 |  |  |
|  |  | 1 | 1.0 |
|  |  | 1 | 4.0 |
|  |  | 1 | missing |
| Trospium | 36 |  |  |
|  |  | 1 | 0.5 |
|  |  | 9 | 15.0 |
|  |  | 2 | 20.0 |
|  |  | 1 | 25.0 |
|  |  | 17 | 30.0 |
|  |  | 2 | 45.0 |
|  |  | 1 | 60.0 |
|  |  | 3 | unknown |
